# A reverse order interview does not aid deception detection regarding intentions

**DOI:** 10.3389/fpsyg.2015.01298

**Published:** 2015-08-31

**Authors:** Elise Fenn, Mollie McGuire, Sara Langben, Iris Blandón-Gitlin

**Affiliations:** ^1^Department of Psychology, Claremont Graduate UniversityClaremont, CA, USA; ^2^Department of Psychology, California State University FullertonFullerton, CA, USA; ^3^Department of Psychology, NorthridgeCA, USA; ^4^Department of Student Affairs Information Systems, University of California RiversideRiverside, CA, USA

**Keywords:** deception detection, Cognitive Load, episodic future thought, Future thinking, investigative interviewing

## Abstract

Promising recent research suggests that more cognitively demanding interviews improve deception detection accuracy. Would these cognitively demanding techniques work in the same way when discriminating between true and false future intentions? In Experiment 1 participants planned to complete a task, but instead were intercepted and interviewed about their intentions. Participants lied or told the truth, and were subjected to high (reverse order) or low (sequential order) cognitive load interviews. Third-party observers watched these interviews and indicated whether they thought the person was lying or telling the truth. Subjecting participants to a reverse compared to sequential interview increased the misidentification rate and the appearance of cognitive load in truth tellers. People lying about false intentions were not better identified. In Experiment 2, a second set of third-party observers rated behavioral cues. Consistent with Experiment 1, truth tellers, but not liars, exhibited more behaviors associated with lying and fewer behaviors associated with truth telling in the reverse than sequential interview. Together these results suggest that certain cognitively demanding interviews may be less useful when interviewing to detect false intentions. Explaining a true intention while under higher cognitive demand places truth tellers at risk of being misclassified. There may be such a thing as too much cognitive load induced by certain techniques

## Introduction

Recent approaches to deception detection focus on exaggerating behavioral differences between liars and truth tellers by overloading a liar’s cognitive resources (Vrij et al., 2011a; Walczyk et al., 2013). These *cognitive load approaches* (CLAs), have been shown to improve deception detection accuracy beyond chance levels (e.g., 60% of observers could accurately identify if someone was lying; Vrij et al., 2008). While these approaches show great promise, the boundaries have not been fully tested (Walczyk et al., 2013; Blandón-Gitlin et al., 2014). In addition, certain conflicting views exist suggesting that lying is not necessarily more cognitively demanding than truth telling (e.g., McCornack, 1997; McCornack et al., 2014), and that the “beyond chance accuracy” is not as robust a finding as initially believed. To date, the majority of studies examining the effectiveness of this approach require participants to lie or tell the truth regarding a past action, such as an autobiographical or episodic event. An equally important goal is to assess whether these new approaches are useful to distinguish between true and false *intent*, a situation in which a target event has not been completed (e.g., a crime not yet committed). This is an important question of significant concern in applied contexts. For example, Transportation Security Administration (TSA) agents must identify a person’s true intentions for boarding a plane, and border patrol officers must identify a person’s true intentions for crossing a border. The purpose of the current study is to assess whether one CLA technique, the reverse order technique, is effective in aiding deception detection when interviewing someone about his or her intentions.

### Cognitive Approach to Deception Detection

A central concept in cognitive deception models is that lying consumes more executive functioning resources (such as working memory, attention, and inhibition) than truth telling (Gombos, 2006; Vrij et al., 2011a; Walczyk et al., 2013 for reviews). Indeed, results of neuroimaging studies (e.g., Abe et al., 2006; Gamer et al., 2007; Christ et al., 2009) and behavioral studies (Walczyk et al., 2005; Debey et al., 2012; Hu et al., 2013; Visu-Petra et al., 2013; Williams et al., 2013) provide support that components of executive functioning underlie deception. For example, behavioral studies suggest that performing a task that interferes with executive processes while answering questions truthfully or deceptively produced greater behavioral differences (e.g., longer reaction times) between liars and truth tellers than not performing the interfering task (Debey et al., 2012; Hu et al., 2013; Visu-Petra et al., 2013). In neuroimaging studies, brain areas (e.g., prefrontal cortex) associated with executive processes are activated to a greater extent during deception than truth telling (Christ et al., 2009). One interpretation of the extant findings is that liars’ behaviors rely on particular executive functioning resources because they must perform multiple tasks, such as suppressing true details, constructing and explaining a lie story, constantly revising and updating the story, all while monitoring the reactions of observers (Walczyk et al., 2009). This is one explanation why lying is more cognitively taxing than truth telling under most conditions.

Cognitive load approaches exploit the already demanding process of lying by making an interview more difficult for liars than truth tellers (Vrij et al., 2011a). One example of how researchers utilize this approach is by asking interviewees to tell their stories in reverse order. Vrij et al. (2008) found that describing a past event in reverse order compared to sequential forward order increased the cognitive load of the interviewees, especially those who were lying. In fact, liars in the reverse order condition were detected more accurately (60% accuracy in lie detection) compared to the sequential forward order interview condition (42% accuracy in lie detection). Police officers also rated liars in the reverse order interview as “thinking harder,” and being more “rigid” and “deliberate” in their responses than liars in the sequential order interview.

Other methods of imposing cognitive load, such as asking participants to recall events in their non-native language (Evans et al., 2013), asking unanticipated interview questions (Vrij et al., 2009), or presenting evidence in a strategic manner [strategic use of evidence (SUE); Hartwig et al., 2006] have exaggerated behavioral differences between liars and truth tellers and increased discrimination accuracy as a result. While CLAs show promise for detecting deception of past actions, the boundaries of the technique needs further exploration (Walczyk et al., 2013; Blandón-Gitlin et al., 2014).

### Detecting True and False Intentions

Most research on CLAs focuses on lying about *past events*, but few research studies test the usefulness of these techniques when interviewing regarding *intentions* (for a review see Granhag and Mac Giolla, 2014). Recalling a past action involves retrieving stored episodic memories from long-term to working memory, whereas explaining future intentions relies on a kind of “mental time travel” whereby a person imagines themselves participating in an event in the future (D’Argembeau and Van der Linden, 2004). This is often referred to as episodic future thinking (EFT), defined by Granhag and Knieps (2011, p. 274) as “the ability to mentally pre-experience one-time personal events that may happen in the future.” Before applying CLA techniques to detect intentions, it is important to understand the cognitive differences between explaining a past action and explaining a true intention (Granhag, 2010). In particular, understanding the differences in cognitive demand involved in recalling a past action compared to imagining a future intention would illuminate whether the underlying assumption (i.e., lying is more demanding than truth telling) of a cognitive load approach was met. If the underlying assumptions were not met (i.e., lying is not more demanding than truth telling regarding intentions) then CLAs would be less effective if used to identify intentions.

Granhag and Knieps (2011) argue that the EFT framework is useful for understanding critical differences between true and false intentions. In support of this, results of some studies suggest that true intentions tend to involve more characteristics of EFT than false intentions (Granhag and Knieps, 2011; Knieps et al., 2013; see also Granhag and Mac Giolla, 2014 for a review). In these studies truth tellers and liars are given 10 min to plan a task (truth tellers) or mock crime (liars), but are intercepted and interviewed before carrying out their plan (referred to as the “Gothenburg Procedure” by Granhag and Mac Giolla). Truth tellers are more likely than liars to report forming a mental image of their task during the planning phase (Granhag and Knieps, 2011; Knieps et al., 2013), which is one characteristic of an episodic future thought.

Behavioral and neuroimaging research on EFT suggest that imagining a true intention is a demanding task on its own. As suggested by Warmelink et al. (2012), results of several studies examining the phenomenological characteristics of imagery for future intentions compared to memory for the past support the idea that imagining the future is demanding (D’Argembeau and Van der Linden, 2004; Gamboz et al., 2010). In these studies, participants imagine an event based on one-word prompts (e.g., “wedding”), and are asked to think about a past memory or future intention (that is likely to happen) related to the prompt (e.g., participants *recall* when they attended a wedding, or *imagine* the next time they will attend a wedding). Participants tend to rate images of the future as less detailed and clear than memories for the past. One explanation for these results is that imagining the future is a cognitively demanding task, drawing on executive processes such as working memory capacity and reducing the ability for participants to imagine the future in detail. In support of this interpretation, Hill and Emery (2013) found that participants’ working memory capacity (a measure of executive processing involved in deception) was a significant predictor for generating specific future episodes. Higher working memory capacity was associated with generating a higher number of specific future episodes. A few studies examining the differences between true and false intentions show similar results: truth tellers and liars provide a similar amount of detail when imagining the future, whereas truth tellers tend to include more detail than liars when recalling the past (Vrij et al., 2011a,b; Warmelink et al., 2012). Further, as discussed in Mac Giolla et al. (2015), holding a true intention may cause more thought-intrusions regarding the future when compared to a false intention. Evidence to support this is cited in Mac Giolla et al. (2015). Truth-tellers had more task-related spontaneous thoughts than liars before completing their intention, further supporting that a genuine intention can demand a high degree of attention even when compared to lying. Taken together behavioral data provide indirect support that imagining a true intention is a cognitively demanding task.

Neuroimaging studies also support that imagining the future involves more cognitive demand than recalling the past. A central argument of Schacter and Addis (2007, p. 773) is that, “since the future is not an exact repetition of the past, simulation of future episodes requires a system that can draw on the past in a manner that flexibly extracts and recombines elements of previous experiences.” That is, imagining a future event involves more cognitive processing steps than remembering the future: imagining the past involves remembering episodic details of a similar past event and then recombining those details to create an imagined and plausible future scenario. Reviews of neurological evidence support this interpretation, such that imagining a future event tends to involve more neural activity than recalling the past (Addis and Schacter, 2012). Taken together, neuroimaging and behavioral data suggest that forming a true intention may be a cognitively demanding task.

### The Present Study

Cognitive load approaches rest on the assumption that liars, but not truth tellers, will be under greater cognitive load when responding to interview questions. Given that imagining a future intention involves additional steps including the retrieval of past memories and constructing a plausible scenario, recalling a true intention may involve similar levels of cognitive load to lying about intentions. Thus, the effectiveness of some CLA techniques may be reduced when trying to discriminate between true and false intent. This study tests this question.

In the present study, we used a demanding cognitive load approach, the reverse order technique, to test whether imposing cognitive load will produce qualitative differences between liars and truth tellers when interviewed about intentions. Liars and truth tellers planned to complete a scenario similar to the procedures of Granhag and Knieps (2011), but were intercepted and interviewed before carrying out their plan. It was predicted that asking liars and truth tellers to explain their intentions in reverse order will induce high levels of cognitive demand that will obscure behavioral differences between these groups and reduce accuracy at detecting deception as a result.

## Experiment 1

Experiment 1 examined whether discrimination accuracy of true and false intent is reduced when the interview involves the reverse order technique compared to a control condition. Third-party observers made truth and lie judgments after viewing truth tellers and liars talking about their intention in reverse order or sequential forward order. The main prediction was that discriminating between truth-tellers and liars would be worse in the reverse than sequential order interview. This is because truth-tellers are hypothesized to experience an increase in cognitive demand in the reverse order interview, and display more deceptive behaviors as a consequence.

### Method

#### Participants and Design

There are two principal parts in this study (1) the creation of stimulus videos from interviews and (2) the collection of third-party observers’ judgments.

##### Part 1

Participants were nineteen students (13 female, 6 male)^1^ from a large university in a metropolitan city. Ten interviewee-participants were in the lie condition and were actively trying to lie about their intentions while the other nine were in the truth condition. Two different cognitive load interviews were used: a low load (sequential forward order interview) and a high load (reverse order interview). There were ten interviewees in the low cognitive load condition (5 in the truth/low and 5 in the lie/low) and nine in the high cognitive load condition (4 in the truth/high and 5 in the lie/high). All interviewees were 18 years of age or older and English-speaking and all completed various measures probing their subjective experiences. The design was a 2 (Veracity: Lie vs. Truth) × 2 [Interview: Sequential (Low Load) vs. Reverse (High Load)] between-subjects design.

##### Part 2

Participants (third-party observers) were recruited through Amazon’s Mechanical Turk (M-Turk) to observe and make true/lie judgments about the interviewees from Part 1. A total of 157 observers watched the 19 interviews and rated whether each person was lying or telling the truth, and how hard each participant appeared to be thinking on a scale of 1 (*Not at all*) to 7 (*Extremely*). As an attention check and to ensure all participants could adequately view the videos, participants were asked an open-ended question about each video at the end of viewing each clip: “Please describe the reason for your response. That is, for what reasons did you decide that this person was lying or truth-telling?” Participants were removed and excluded from all analyses if they reported difficulty viewing or seeing the person when answering this question. Participants were also removed if they did not respond to these questions or wrote nonsense responses (such as numbers). Participants were also excluded if they took the survey more than once (as indexed by their IP address). Approximately 16 observers were excluded, leaving a total of 145 observers in the remaining analyses. A total of 145 observers were used in the remaining analyses. Approximately 43% of these observers were males, with ages ranging from 18 to 72 years of age (*M* = 35, *SD* = 12.83). A majority of observers reported having a bachelor’s degree (41.4%), with the remaining observers reported some college (23%), a post-graduate degree (12%), an associate degree (12%) or high school or below (11%). A majority of participants reported ethnicity of Caucasian (75%) with the remaining participants reported Asian (9%), Black or African American (6%), Hispanic (6%), Native American (1%) or mixed (4%). An initial screening via options available through the survey provider ensured that only participants in the United States could participate. This was a 2 (Veracity: Lie vs. Truth) × 2 (Interview: Reverse vs. Sequential) within-subjects design.

#### Materials and Procedure

Part 1 included several phases that were designed based on procedures from Granhag and Knieps (2011). First, each interviewee was greeted, given an informed consent, and then asked to plan and elaborate on a task during the *planning phase*. Next, each interviewee was sent to carry out the task they planned but was intercepted and asked to answer some questions about their intentions during the *interview phase*. After the interview, each interviewee completed a follow-up questionnaire during the *post-interview questionnaire phase.*

##### Planning phase

After completing an informed consent form, participant-interviewees were given instructions to plan and subsequently carry out a task; they were told that they had 10 min to plan and 15 min to carry out the task. Truth tellers were asked to find reference materials in the library on a government topic (one periodical and one book). The library was chosen because it is a moderately familiar place to all participants and is large enough to ensure that participants would need to plan their task considering the relevant periodicals and books they were to get were on separate floors of the library; these criteria were chosen based on Granhag and Knieps (2011) methods. The researchers expected that this instruction would encourage participants to plan an efficient route and form a concrete plan because of the wide spatial area they needed to cover in a short amount of time.

Liars received similar instructions and time frame as truth tellers, except that they were told to steal the reference materials from the library and were suggested to use some of their time planning a cover story to conceal their true intentions if intercepted. These instructions are based on the methodology of Granhag and Knieps (2011), although the library task was novel in the present experiment.

To assist with planning, each participant was provided with a computer and given a link to the map of the library, a hard copy of this map, and a link to the library website. At the end of the 10 min, the participant was escorted out of the room in which the planning phase took place and asked, “What are you doing next?” to ensure that the participant intended to go to the library.

##### Interview phase

After the planning phase, the participant was intercepted by a confederate before reaching the library. The participant was escorted to an interview room and asked to wait for an interviewer. Before being interviewed the researcher who was with them during the planning phase was escorted into the interview room as if they had been asked to answer questions as well. The researcher told the participant to be as convincing as possible (truth tellers were told to “just be honest” and liars were reminded to use their cover story and not reveal their actual plans). The interviewer then dismissed the researcher.

All interviews were video-recorded. The participant was randomly assigned to an interview approach condition that was either low or high in cognitive load. The interviewer was blind to the veracity condition. In the low load condition, participants were asked to describe their future intentions using a sequential order interview procedure. In the high load condition participants were asked to describe their future intentions using a reverse order interview procedure. These instructions were based on the interview instructions from Vrij et al. (2008), with slight modifications to match the planned library task and to ensure that participants were recalling their future intention rather than a past action. Participants in the sequential order condition were given the following instructions:

“*I want you to tell me everything that you were going to do in the 15 min following from when you were intercepted. Therefore, you should start your story with the first place that you intended to be when you were intercepted, and end your story with the last place you intended to be at the end of those 15 min.*”

Participants in the reverse order were given the following instructions:

“*I want you to tell me everything that you were going to do in the 15 min following from when you were intercepted, but in reverse order. Therefore, you should start your story with the last place you intended to be at the end of those 15 min, and end your story with where you were when you were intercepted.*”

##### Post-interview questionnaire phase

Immediately after the interview, the participants were escorted back to the planning room and told that the role-playing part of the experiment was over and they should fill out the post-interview survey as honestly as possible. This questionnaire was designed to assess the subjective experiences of the participant during the interview. **Tables 1** and **2** provide the set of questions used. As a manipulation check each interviewee rated the extent to which they were lying or telling the truth during the interview on a scale from 1 (*Everything I said was a lie*) to 7 (*Everything I said was the truth*). In addition, participants rated the degree of motivation and realism they experienced on 1 (Not at all) to 7 (Extremely) scales. As a manipulation check for the cognitive load condition, participants rated, “Overall, how easy or difficult did you find the interview?” on a scale from 1 (Extremely Easy) to 7 (Extremely Difficult). Participants also rated their performance during the interview on 1 (Not at all) to 7 (Extremely) scales for how convincing they perceived themselves to be, and how nervous they were during the interview. Participants reflected on the difficulty of answering one of the interview questions: “Please rate how easy or difficult it was for you to answer the following question during the interview: ‘Did you, at any point during your planning, evoke a mental image of the future event?’ on a scale from 1 (Extremely Easy) to 7 (Extremely Difficult). Finally, participants were asked, “To what extent did you form a mental image of your errand during the planning phase?” on a scale from 1 (A very low extent) to 7 (A very high extent), along with several questions designed to assess the clarity and perceptual details of the mental image formed (i.e., visual, auditory, olfactory, gustatory, spatial, and sequential details) on scales from 1 (A very low extent) to 7 (A very high extent). These questions, adapted from Granhag and Knieps (2011), were designed to address differences in the cognitive processes involved in imagining an intention compared to a cover story. Several other questions were asked as part of another study, participants were debriefed, and course credit was provided in exchange for their participation.

**Table 1:** Ratings of motivation and realism.

	Truth tellers				Liars			
	Sequential		Reverse		Sequential		Reverse	
	*M*	*SD*	*M*	*SD*	*M*	*SD*	*M*	*SD*
*To what extent were you motivated to do the study?*	5.60	1.34	5.25	0.96	5.40	1.14	4.80	0.84
*To what extent did you expect to be interviewed about your intentions?*	4.20	1.92	2.25	0.96	2.80	1.79	5.20	1.48
*To what extent did you feel like you were a suspect being interviewed in a real-life criminal interview?*	3.80	1.10	5.00	0.82	4.40	1.14	5.60	1.14

**Table 2:** Ratings of subjective experience of psychological processes.

	Truth tellers				Liars			
	Sequential		Reverse		Sequential		Reverse	
	*M*	*SD*	*M*	*SD*	*M*	*SD*	*M*	*SD*
**Cognitive load**								
*Overall, how easy or difficult did you find the interview?*	2.20	1.10	3.75	2.06	3.40	1.82	3.60	1.14
**Convincing**								
*How convincing do you think you were during the interview?*	4.80	1.92	3.50	1.73	4.20	1.10	4.80	2.39
**Nervousness**								
*How nervous did you feel during the interview?*	3.20	1.48	5.00	1.41	4.80	2.17	4.80	1.79
**Mental imagery**								
*Please rate how easy or difficult it was for you to answer the following question during the interview: “Did you, at any point during your planning, evoke a mental image of the future event?”*	3.20	0.84	3.75	0.96	4.80	1.64	4.00	1.58
To what extent did you form a mental image of your errand during the planning phase?*	5.60	1.14	5.25	1.71	4.60	0.89	6.25	0.96
To what extent was your mental image characterized by…*								
*Visual information?*	5.80	0.84	5.75	0.96	5.60	0.89	6.00	1.41
*Auditory information?*	3.40	2.07	2.00	0.82	3.00	1.41	5.00	1.63
*Smell?*	1.00	0.00	1.00	0.00	1.40	0.55	2.00	1.15
*Taste?*	2.20	2.68	1.00	0.00	1.40	0.55	1.75	0.96
To what extent was your mental image characterized by clarity with respect to…*								
*Where objects were located in your mental image?*	6.20	0.84	5.00	1.41	5.20	0.84	4.50	2.65
*Where people were located in your mental image?*	4.40	1.82	2.75	1.50	4.60	2.07	6.00	0.82
*The following of one event after another in time?*	5.00	1.00	4.25	0.96	4.40	1.82	5.50	1.29
*To sum up your experience during planning phase how clearly did you imagine yourself doing the future event?*	5.80	1.64	4.75	1.50	4.20	1.48	4.75	2.22

*Liars were asked to describe their cover story; truth tellers were asked to describe their intention.

*One truth-teller in the sequential order condition did not respond to the mental imagery questions.

##### Part 2

This part involved the judgment component. Third-party observers were recruited through Amazon’s M-Turk and compensated $.75 in exchange for their participation. Data from M-Turk subjects can be comparable to laboratory subjects, and the M-Turk sample tends to be somewhat more diverse than an undergraduate college sample (Buhrmester et al., 2011). Observers completed a survey via Qualtrics in which they watched all of the 19 video-recorded interviews. The mean duration of videos was 52.84 s (*SD* = 8.92), ranging from 40 to 69 s. Participants indicated “yes” or “no” whether each person was lying or telling the truth, and how hard each participant appeared to be thinking on a scale of 1 (*Not at all*) to 7 (*Extremely*); several other questions were asked as part of another study.

### Results and Discussion

Prior to the main analyses, univariate and multivariate screening procedures were performed on both the interviewee (*n* = 19) and observer (*n* = 145) data sets. The 19 interviewee participants as well as all observer participants were kept for the analyses.

#### Manipulation Checks^2^

Means and *SD* are reported in **Tables 1** and **2**. For all manipulation check questions. Notable differences between conditions are described below.

##### The lying condition

Liars lied more during the interview (*M* = 3.00, *SD* = 1.76, *n* = 10) and truth tellers were more likely to tell the truth (*M* = 6.67, *SD* = 0.71, *n* = 9) Cohen’s *d* = 2.68.

##### Motivation and realism

Participants in the reverse order (*M* = 5.3, *SD* = 1.0) reported experiencing more realism than in the sequential order condition (*M* = 4.1, *SD* = -1.1). Overall, participants were motivated to participate in the study (*M* = 5.26, *SD* = 1.05) and felt like they were real suspects being interviewed (*M* = 4.68, *SD* = 1.20). **Table 1** includes means and *SD* for these questions.

##### Engagement in the planning phase

Participants rated how difficult the planning phase was on a scale from 1 (*Not at all difficult*) to 7 (*Very difficult*). Overall, participants reported low levels of difficulty during the planning phase [*M* = 3.00, *SD* = 1.2, on a scale of 1 (*Not at all*) to 7 (*Very*)]. Liars (*M* = 3.80, *SD* = 1.69) reported more preparation for the interview than truth tellers (*M* = 2.33, *SD* = 1.12).

##### Subjective experience of psychological processes

**Table 2** reports these means and *SD* of these questions.

In sum, these manipulation checks show the expected results; participants followed instructions, and were equally motivated and engaged in the tasks.

#### Observers’ Judgments

##### Accurate identifications

**FIGURE 1:**
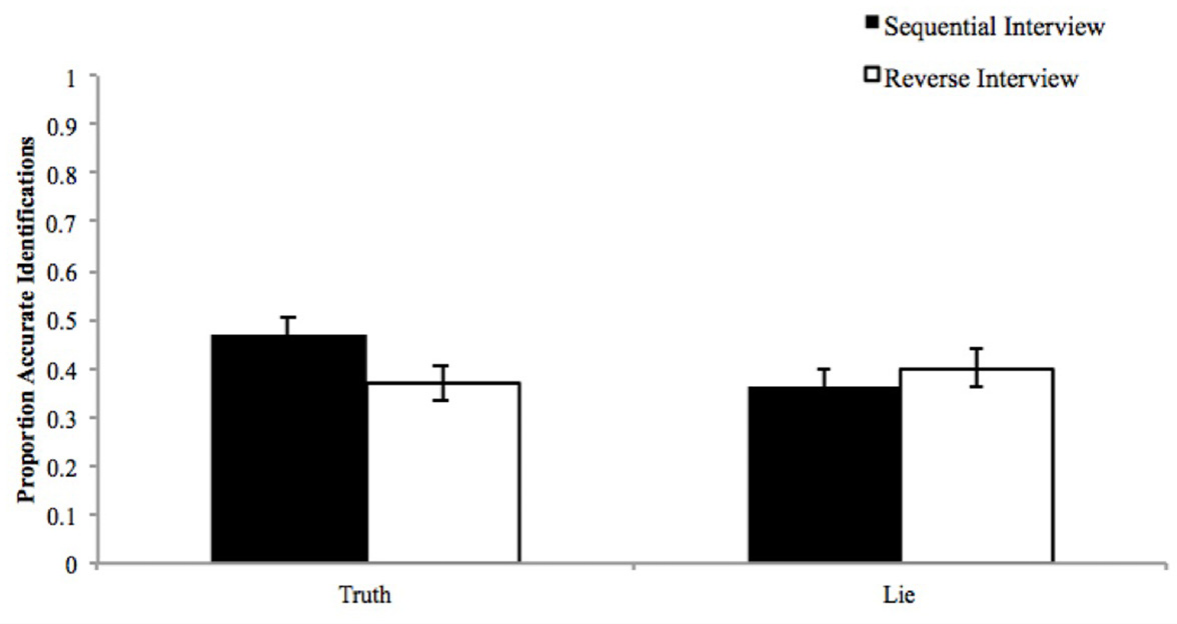
**The proportion of accurate identifications as a function of veracity and interview condition.** An accurate true identification implies that observers responded “true” when the participant was telling the truth. An accurate lie identification implies that observers responded “lie” when the participant was telling a lie.

Third-party observers’ proportion correct for liars (*n* = 10) and truth tellers (*n* = 9) data were analyzed using a 2 (Veracity: Lie vs. Truth) × 2 (Interview: Reverse vs. Sequential) within-subjects ANOVA. There was a significant interaction between veracity and interview, as displayed in **Figure 1**, *F*(1,144) = 14.54, *p* < 0.001, such that observers identified truth tellers (*M* = 0.47, *SD* = 0.25) more accurately than liars (*M* = 0.36, *SD* = 0.21) in the sequential interview; *t*(144) = 3.63, *p* < 0.001, *d* = 0.48 whereas observers correctly identified liars (*M* = 0.40, *SD* = 0.24) and truth tellers (*M* = 0.37, *SD* = 0.26) at about the same rate in the reverse order interview, *t*(144) = 1.12, *p* = 0.265, *d* = 0.12. Further, and more critically, observers were more accurate at identifying truth tellers in the sequential than reverse order condition, *t*(144) = 3.68, *p* < 0.001, *d* = 0.39 whereas liars were marginally better identified in the reverse than sequential order condition, *t*(144) = 1.81, *p* = 0.073, *d* = 0.18. While these results show differences among groups, it is important to note that these means are all below chance levels (0.50). This suggests an overall difficulty in assessing veracity of future intent accounts, which involve abstractions of events that have not occurred. See **Figure 1** for a depiction of this interaction.

##### Signal detection analysis

The non-parametric signal detection measures of *A*′ and *B*″ were computed to assess discrimination accuracy independent of bias. *A*′ measures discrimination accuracy and is an alternative to *d*′ when used with forced choice answers. Observers’ discrimination accuracy did not significantly differ between sequential (*M* = 0.38, *SD* = 0.20) and reverse order (*M* = 0.35, *SD* = 0.21) interview, *t*(144) = 1.22, *p* = 0.226, *d* = 0.15. However there were significant differences in response bias as measured by *B*″, such that observers were more biased to say “lie” when viewing interviews in the reverse (*M* = -0.06, *SD* = 0.31), than in the sequential order interview (*M* = 0.05, *SD* = 0.27), *t*(144) = 3.45, *p* = 0.001, *d* = 0.38. *B*″ is a measure of response bias (Stanislaw and Todorov, 1999) and in this context it ranges from -1 (extreme “lie” bias) to 1(extreme “truth” bias), with values of 0 indicating no bias. In the reverse (*p* = 0.026) and sequential (*p* = 0.016) order condition, bias differed significantly from zero. This suggests that there was a significant bias toward responding “lie” in the reverse order condition, and a significant bias toward responding “truth” in the sequential order condition.

##### Objective assessment of cognitive load

To assess for perceived cognitive load, observers rated how hard the participant appear to be thinking, from 1 (*Not hard at all*) to 7 (*Extremely hard*). A 2 (Veracity: Lie vs. Truth) × 2 (Interview: Reverse vs. Sequential) within-subjects ANOVA detected significant effects of veracity, interview and an interaction. Truth tellers (*M* = 4.34, *SD* = 0.93) were rated as experiencing more cognitive load than liars (*M* = 3.73, *SD* = 0.92), *F*(1,144) = 87.77, *p* < 0.001, *d* = 0.66. Participants in the reverse order interview (*M* = 4.28, *SD* = 0.88) were rated as experiencing more cognitive load than in the sequential order interview (*M* = 3.80, *SD* = 0.93), *F*(1,144) = 72.67, *p* < 0.001, *d* = 0.53. A significant interaction between veracity and interview was also detected, *F*(1,144) = 18.15, *p* < 0.001. While both truth tellers and liars were rated as experiencing more load in the reverse than sequential order interview, the magnitude of these differences was greater for truth tellers, [(Reverse: *M* = 4.69, *SD* = 1.06); (Sequential: *M* = 4.0, *SD* = 1.05) *t*(144) = 8.46, *p* < 0.001, *d* = 0.65], than liars [(Reverse: *M* = 3.86, *SD* = 0.99); (Sequential: *M* = 3.59, *SD* = 1.02), *t*(144) = 4.06, *p* < 0.001, *d* = 0.27].

The prediction in Experiment 1 was partially supported; those telling the truth about their future intent were less accurately identified in the reverse order condition compared to the sequential order condition. On the other hand, those lying about their intent were (marginally) better detected in the reverse than sequential order condition. Results of the signal detection measure *A*′ partially support hypotheses such that observer accuracy in the reverse order interview was similar when compared to the sequential order interview. Further, inducing cognitive load shifted response bias, yielding more judgments of deception (rather than truth). These results may be explained by observers’ reports of interviewees’ high cognitive load displays in the reverse order condition (especially by truth tellers), compared to the sequential order condition. A shift in response bias is interesting because it suggests that in general, observers are responding more liberally (adopting a “respond lie” response bias) when viewing videos of interviewees experiencing higher levels of cognitive load (the reverse, rather than sequential interview), regardless of interviewee’s actual veracity. These results support the hypothesis that when describing an intention, liars and truth-tellers experiences are more similar, and cause them to produce similar behavioral appearances (in this instance, cognitive load and anxious appearances). In essence, the pattern of results suggest that truth tellers in the reverse order condition looked more like liars as a result of increased cognitive load. These findings fit with a new line of literature suggesting that certain cognitive load manipulations are simply too cognitively demanding – both liars and truth-tellers experience high levels of cognitive load because of the manipulation and therefore appear similar to one another (Blandón-Gitlin et al., 2014). To examine this interpretation further, Experiment 2 was conducted. The extent to which truth tellers and liars displayed cues to deception and truthfulness were assessed.

## Experiment 2

Given that in the reverse order condition in Experiment 1 truth tellers were at a disadvantage in terms of being identified correctly and observers showed a lie bias, what interview behaviors could have been revealed in this condition? In Experiment 2 a new set of third-party observers were asked to determine the extent to which interviewees in the various conditions displayed signs of specific behaviors. These observers were experiment-blind as to the purpose of the study. Thus, assessing deception was never part of these observers’ evaluation. Based on the results of Experiment 1, it was predicted that more behavioral cues to deception and fewer signs of truthfulness would be evident in the reverse order condition than the sequential order condition. Moreover, this would be especially the case for truth tellers discussing their true intent.

### Method

#### Participants and Design

A total of 91 observers watched the 19 interviews and rated 10 behavioral cues. Participants were removed and excluded from all analyses if they reported difficulty viewing or seeing the person when answering this question. As an attention check, participants were excluded if they did not respond to these questions or wrote nonsense responses (such as numbers), or if they took the survey more than once (as indexed by their IP address). Approximately seven observers were excluded, leaving a total of 84 observers in the remaining analyses approximately 49% of the 84 observers were males, with ages ranging from 21 to 67 years (*M* = 34.90, *SD* = 11.73). A majority of observers reported having a bachelor’s degree (42%), with the remaining observers reporting some college (26%), a post-graduate degree (8.3%), an associate degree (10%), or high school or less (14%). A majority of observers reported Caucasian ethnicity (not Hispanic) (74%), with the remaining participants reporting Black or African American (11%), Asian (10%) or Hispanic (6%) ethnicity. Only participants in the United States were allowed to participate. These observers were independent, and did not participate in Experiment 1. All 19 videos from each condition were shown an equal number of times. The design is the same as in Experiment 1, a 2 (Veracity: Lie vs. Truth) × 2 (Interview: Reverse vs. Sequential) within-subjects design.

#### Materials and Procedure

Third-party observers were recruited through M-Turk to complete a survey on Qualtrics. They first reviewed an informed consent and then asked if they would like to participate. They were asked to watch the 19 interviews and rate 10 behavioral cues (e.g., how hard does the interviewee appear to be thinking?) on scales from 1 (*Not at all*) to 7 (*Extremely*) after each video. The four cues used in the current experiment (i.e., cognitive load, anxiety, confident and convincing appearance, and control behavior) were chosen based on results of Hartwig and Bond (2011), along with other comprehensive meta-analyses (e.g., DePaulo et al., 2003; Hauch et al., 2014). For example, Hartwig and Bond (2011) meta-analyzed the relationship between the appearance of lie and truth behavioral cues and actual veracity across 134 cues. In addition to other cues, their results suggested that liars appeared to be thinking harder, more indifferent, and less spontaneous than truth-tellers. Truth-tellers appeared to be more cooperative and relaxed, and produced stories that were more realistic than liars. All questions used are listed in **Table 3**. Finally, third-party observers were debriefed and paid $0.75 for their participation.

**Table 3:** Ratings for behavioral cues to deception as a function of interview type and veracity.

	Truth tellers						Liars					
	Sequential		Reverse				Sequential		Reverse			
Behavioral cues of deception	*M*	*SD*	*M*	*SD*	*p*	*d*	*M*	*SD*	*M*	*SD*	*p*	*d*
**Cognitive demand**	**3.82**	**1.32**	**4.58**	**1.29**	**<0.001**	**0.58**	**3.31**	**1.22**	**3.36**	**1.2**	**0.776**	**0.04**
How hard does the interviewee appear to be thinking?*	4.1	1.53	5.06	1.5	<0.001	0.63	3.57	1.58	3.79	1.47	0.314	0.14
*How much difficulty does the interviewee appear to be having in answering the questions?*	3.96	1.78	4.81	1.71	0.002	0.46	3.31	1.67	3.46	1.61	0.537	0.09
*How distracted does the interviewee appear?*	3.38	1.65	3.88	1.84	0.039	0.29	3.04	1.77	2.83	1.5	0.382	0.13
**Anxiousness**	**3.71**	**1.38**	**4.34**	**1.57**	**0.007**	**0.43**	**3.28**	**1.54**	**3.24**	**1.35**	**0.852**	**0.03**
*How anxious does the interviewee appear?*	4.13	1.6	4.63	1.63	0.058	0.31	3.23	1.67	3.27	1.5	0.847	0.03
*How much does the interviewee appear to fidget?*	3.29	1.68	4.05	1.84	0.005	0.43	3.33	1.66	3.2	1.57	0.603	0.08
**Confident and convincing appearance**	**3.59**	**1.21**	**3.24**	**1.34**	**0.071**	**0.27**	**4.14**	**1.38**	**3.87**	**1.28**	**0.195**	**0.2**
*How detailed are the interviewee’s responses?*	4.1	1.5	3.62	1.76	0.049	0.29	4.31	1.64	3.62	1.64	0.01	0.42
*How spontaneous is the interviewee?*	3.25	1.4	3.3	1.57	0.797	0.03	3.82	1.63	3.79	1.5	0.868	0.02
*How confident does the interviewee appear?*	3.25	1.61	2.83	1.57	0.1	0.26	4.27	1.65	3.92	1.72	0.171	0.21
*How convincing is the interviewee?*	3.77	1.7	3.2	1.68	0.023	0.34	4.15	1.83	4.17	1.54	0.965	0.01
**Controlling**												
*How controlled is the interviewee’s posture?*	4.06	1.65	3.95	1.63	0.684	0.07	4.45	1.43	4.05	1.54	0.102	0.27

*There were 84 observers in total. However, there was one missing response to the “Thinking Hard” item (for truth tellers only) and “Difficulty” item (for liars only).

### Results and Discussion

#### Behavioral Cue Differences among Groups

For simplicity, nine of the ten associated behavioral cues ratings from third-party observers were averaged to create three variables: cognitive load, anxiety, and confident and convincing appearance^3^. **Table 3** presents average ratings for each item per condition. A 2 (Veracity: Lie vs. Truth) × 2 (Interview: Reverse vs. Sequential) within-subjects ANOVA was run for each variable.

##### Cognitive load

Three questions were averaged to assess perceived cognitive load appearance (all cues to deception, Cronbach’s α = 0.75). There was a main effect of veracity and order, and a significant interaction between veracity and order. Observers rated truth tellers (*M* = 4.20, *SD* = 1.30) as experiencing significantly more cognitive load than liars (*M* = 3.33, *SD* = 1.21), *F*(1,83) = 44.77, *p* < 0.001, *d* = 0.69. Observers rated interviewees in the reverse order condition (*M* = 3.97, *SD* = 1.25) as experiencing more cognitive load than the sequential order condition (*M* = 3.57, *SD* = 1.27), *F*(1,83) = 9.45, *p* = 0.003, *d* = 0.32. The significant veracity by interview order interaction *F*(183) = 9.94, *p* = 0.002 shows that truth tellers appeared to experience significantly more cognitive load in the reverse order interview (*M* = 4.58, *SD* = 1.29,) than the sequential order interview (*M* = 3.82, *SD* = 1.32), *t*(83) = 4.25, *p* < 0.001, *d* = 0.58, whereas there was no significant difference for liars, *p* = 0.776. These results are similar to those from Experiment 1 where a different set of observers judged the displays of cognitive load appearance in interviewees. This result represents a robust effect.

##### Anxiety

Two questions were averaged to create the variable anxiety (both cues to deception, Cronbach’s α = 0.80). There was a main effect of veracity, and a significant interaction between veracity and interview. Observers rated truth tellers (*M* = 4.02, *SD* = 1.47) as more anxious than liars (*M* = 3.26, *SD* = 1.44), *F*(1,83) = 29.93, *p* < 0.001, *d* = 0.52. There was also a significant interaction *F*(1,83) = 4.99, *p* = 0.028, whereas truth tellers appeared to be more anxious in the reverse order interview (*M* = 4.34, *SD* = 1.57) than the sequential order condition (*M* = 3.71, *SD* = 1.37, *t*(83) = 2.77, *p* = 0.007, *d* = 0.43, there was no significant difference for liars, *t*(83) = 0.19, *p* = 0.852, *d* = 0.03.

##### Confident and Convincing

Four questions were averaged to create the variable called confident and convincing (all cues to truthfulness, Cronbach’s α = 0.82). There was a main effect of veracity and order, but not an interaction. Observers rated liars (*M* = 4.00, *SD* = 1.33) as more confident and convincing than truth tellers (*M* = 3.42, *SD* = 1.28), *F*(1,83) = 21.53, *p* < 0.001, *d* = 0.44. Observers rated interviewees in the sequential interview condition (*M* = 3.87, *SD* = 1.30) as more confident and convincing than interviewees in the reverse order condition (*M* = 3.55, *SD* = 1.31), *F*(1,83) = 4.67, *p* = 0.033, *d* = 0.35.

##### Control

Observers also rated the degree of control displayed by each interviewee, a cue to deception. There were no significant effects on this question (*F*s < 3.51).

In Experiment 2 the prediction that more behavioral cues associated with lying and fewer signs of truthfulness would be evident in the reverse than the sequential order condition was confirmed with measures of cognitive load and confident-convincing appearance. More importantly, the second prediction (that this effect would be especially the case for interviewees discussing their true intent) was confirmed with measures of cognitive load, anxiety, and confident-convincing appearance. These results support those of Experiment 1 suggesting that because truth tellers showed more behavioral signs of deception and fewer signs of truthfulness, accuracy in identifying them correctly was reduced in the reverse order interview. Surprisingly, truth-tellers displayed more behavioral cues than liars in the reverse order condition. These results suggest that truth tellers are actually more cognitively loaded than liars when a CLA technique was used. Together these results support that a CLA approach such as the reverse order interview may not be useful for discriminating between true and false intentions: truth-tellers looked more like liars, whereas liars displayed similar behaviors regardless of interview technique.

## General Discussion

This study investigated the applicability of a cognitively demanding interview as a viable option to detect deceptive intentions. Specifically, this study tested whether subjecting individuals to the reverse order interview technique (a high cognitive load interview) would enhance deception detection accuracy compared to a sequential order interview technique (a low cognitive load interview). There were three principal findings. First, an interaction was detected between veracity and interview order such that the reverse compared to sequential order interview condition lowered accurate identifications for truth tellers more than liars. Second, third-party observers rated those in the reverse order interview as thinking harder (experiencing more cognitive demand) than the sequential interview; the magnitude of this difference was greater for truth tellers than liars. Third, truth tellers exhibited more behavioral cues indicative of deception and less behavioral cues indicative of truth during the reverse, but not sequential interview condition.

Why did a reverse order technique fail to increase accuracy at discriminating between liars and truth-tellers? Answering questions regarding one’s true intent may be a cognitively demanding task. This is why observers’ had a bias to call all interviewees “liars” when viewing reverse-order interviews. Further and more surprising was the fact that the cognitively demanding, reverse order technique impacted truth tellers more than liars: two independent groups of third-party observers rated truth tellers as thinking harder in the reverse than sequential order interview, and the magnitude of this difference was greater when compared to liars. The CLAs are intended to overload liars more than truth tellers because lying typically requires more cognitive resources than truth telling. In this study, truth tellers may have experienced similar (or greater) levels of cognitive demand compared to liars. As a consequence, truth tellers were misidentified more in the reverse than sequential order condition whereas liars were identified at about the same rate in both interviews.

Why did truth-tellers experience an increase in deceptive behaviors, even more so than liars? One explanation for this is that liars and truth-tellers utilize unique strategies when responding to questions, and these strategies rely on cognitive processes such as executive functioning to different degrees. Research on past actions suggests that truth-tellers’ strategies typically involve simply telling the truth. This is because truth-tellers tend to believe that their truth will be apparent to observers, thus just explaining their intention as they expected the events to occur would suffice. If this strategy holds true for intentions, explaining a true intention may be an especially demanding task that relies heavily on executive processes. As a consequence, when completing a difficult interview task such as explaining an event in reverse order, truth-tellers’ ability to appear truthful may be reduced. Indeed, research on EFT suggests that envisaging oneself carrying out future events involves multiple information processing steps that rely on executive processing, such as recombining past experiences with future intended actions, choosing plausible temporal-spatial coordinates to imagine the intended actions, and combining these and other steps together to create the whole intention (D’Argembeau et al., 2010; de Vito et al., 2012). Truth-tellers in this study explained an intention that involves constructing an image of themselves carrying out a series of activities that are novel and then reporting on that event. Thus, it is hypothesized that the additional cognitive demand associated with EFT reduced the truth teller’s capacity to appear truthful when placed in a demanding situation (i.e., reverse order interview).

Liars, however, may have relied on strategies that avoid the difficulty involved in EFT. For example, one liar explained post-interview that she answered her interview questions by describing a habitual activity (walking through the library to go to basketball practice) that she performed every week during the same time. Explaining this schema-like memory may have been less effortful than explaining a true, detailed intention about a novel series of events. Future research should consider how strategies impact the effectiveness of CLA techniques, and consider the specific cognitive processes involved.

### Low Accuracy Rates

Why were observers so abysmal (below chance levels in Experiment 1) at discriminating between liars and truth-tellers? A widely cited meta-analysis (Bond and DePaulo, 2006) suggests that deception is detected at just above chance levels (i.e., 54%); the accuracy rates in this study are not even close. Unfortunately, there is no meta-analytic data on the accuracy of detecting true and false intentions so it is unclear what the accuracy rate is for detecting a true or false intention. In addition, the studies that have examined accuracy at discriminating between true and false intentions do not typically utilize human observers for computing discrimination accuracy between true and false intentions. Only two studies that the authors are aware of have used human observers. In Warmelink et al. (2011), the accuracy data is reported from *n* = 2 observers who detected 76% of truth tellers and 77% of liars. The second study by Vrij et al. (2011b) asked observers to read transcripts of interviews, and showed an accuracy rate of about 70%. The conclusions that can be drawn regarding general accuracy at discriminating between true and false intentions based on only these two studies are limited. In Warmelink et al. (2011), only two observers were used. In addition, these interviewers asked 11 questions about different topics related to the senders’ intentions. As suggested by research from Masip et al. (2009), Street and Masip (2015), observers (interviewers) often improve their deception detection accuracy during the course of an interview when using multiple compared to single questions because of the improved ability to evaluate senders’ consistency to different questions. This may be another explanation for the increased accuracy of the two interviewers in Warmelink et al. (2011) when compared to our study. Based on the limited availability of data regarding observer accuracy discriminating between true and false intentions, it is unclear what the average accuracy rate is discriminating between true and false intentions and therefore not necessarily unusual that our accuracy rates are low. Instead, it is possible that discriminating between true and false intent is indeed difficult under certain circumstances as in this study.

### Relationship to Other Cognitive Lie Detection Approaches

The current study provides preliminary evidence that a reverse order technique may not be useful when interviewing someone about his or her intentions. However, results of at least one study suggest lying about an intention is more cognitively demanding than telling the truth, similar to research on past actions (Suchotzki et al., 2015). Suchotzki et al. (2015) found that lying about a past action or future intention produced longer RTs than telling the truth, suggesting that there is a higher cognitive cost to telling a false than true intention. However, the interview task was an adaptation of the Scheffield Lie Test, a “yes/no” paradigm that may be less desirable in situations where gathering intelligence is necessary, and likely demands less cognitive resources from an interviewee to appear truthful than answering open-ended questions during a CLA.

Results of other studies reveal mixed results regarding the effectiveness of CLAs for detecting intentions. For example, the SUE technique was effective whereas using unanticipated questions depends on the type of questions asked. Some studies suggest that asking unanticipated questions about the past (inquire about the activities while planning an intention, such as details about rental cars or hotel reservations when traveling), or presenting evidence strategically about the past, are effective techniques for enhancing differences between liars and truth tellers (Clemens et al., 2011; Sooniste et al., 2013). Results of another study suggest that only certain unanticipated questions (i.e., inquiring about transportation plans) are effective at eliciting differences between liars and truth tellers (Warmelink et al., 2012). Further, asking unanticipated questions about planning in small groups of suspects showing mixed results. In one study, the difference between liars and truth-tellers on measures such as within-group consistency in small groups of suspects were similar when asking unanticipated or anticipated questions (Mac Giolla and Granhag, 2014). However, in another study truth-tellers showed higher within-group consistency levels than liars for unanticipated but not anticipated questions (Sooniste et al., 2014).

It is still unclear what mechanisms underlie these various types of CLAs (Blandón-Gitlin et al., 2014). What is clear from results of previous studies and the current study is that some categories of CLAs may be more or less useful with identifying intentions. The effectiveness of such approaches may depend on the interaction between the demands of the imposed load technique and the mental processes involved. Future research should focus on these interactions, investigating cognitive mechanisms and boundary conditions of these techniques so that CLAs can be calibrated and applied appropriately for use in the field

It is important to note that the sender sample, which makes up the stimulus materials in this study was relatively small (10 liars and 9 truth-tellers). Although not atypical in recent lie detection research (e.g., Blair et al., 2010; Leal et al., 2010; Evans et al., 2013) this could be a limitation in generalizing our results. In a recent meta-analysis investigating multiple cues and various artifacts affecting accuracy, it was revealed that out of 144 studies the average number of senders per study was 64 (Hartwig and Bond, 2011). This same meta-analysis, however, showed that lie detection was not significantly related to the number of senders. Additionally in a recent reverse order study by Evans et al. (2014) eight total videos (four in a reverse order and four in a chronological order interview) of true and false alibi statements were used. Thus, our procedure is in line with recent studies and findings should not reflect an artifact of our stimulus materials.

## Conclusion

This study shows the complexities of using a cognitive load approach, especially when examining intentions. The results of this study suggest that in certain situations, such as explaining an intention, liars and truth tellers may experience similar levels of demand. These situations may reduce the effectiveness of the cognitive load approach by increasing the number of false accusations of truth tellers. Certain CLA techniques such as the reverse order interview may be ineffective for detecting deceptive intentions, mainly because the reverse order interview makes it difficult to appear honest. Put another way, there may be such a thing as too much cognitive load, especially for truth tellers. Practitioners attempting to apply new cognitive load techniques to interview suspects must take these finding into account to protect innocent individuals from being misclassified.

## Conflict of Interest Statement

The authors declare that the research was conducted in the absence of any commercial or financial relationships that could be construed as a potential conflict of interest.

## Footnotes

1 A total of 22 interviews were conducted. However, two clips were deleted because the auditory or visual data were damaged, and one participant was deleted because they failed to follow the interview instructions.

2 Descriptive statistics were used exclusively as requested by a reviewer.

3 The cluster variables were created based on the face validity of the nine items. Factor analyses were not appropriate given the number of variables and sample size (Tabachnick and Fidell, 2012). The Cronbach’s alpha values for the three clusters were >0.75. The one question on controlled appearance was analyzed separately, as it was not strongly associated with the items in the other variables. Note on **Table 3** that the means on each question show similar patterns as those of the clustered variables.
